# Evaluation of Antiarthritic Potential of Methanolic Extract of *Gentiana kurroo* Royle

**DOI:** 10.1155/2014/810615

**Published:** 2014-11-06

**Authors:** Khan Mubashir, Bashir A. Ganai, Khalid Ghazanfar, Seema Akbar

**Affiliations:** ^1^Department of Biochemistry, University of Kashmir, Srinagar 190006, India; ^2^Regional Research Institute of Unani Medicine, Kashmir University Campus, Srinagar 190006, India

## Abstract

Rheumatoid arthritis is a systemic disorder which involves the activation of immune system against the self-tissues. The main targets of this disease are the joints. Being systemic the development of this disease involves different mechanisms and thus the exact cause of this disease remains unknown. Although different drugs have been developed, none has been found to be the cure for this disease. In the current study the rat carrageenin paw was used as a model for acute inflammation and mycobacterium induced adjuvant arthritic model was used for exploring the antiarthritic potential of methanolic extract of *Gentiana kurroo*. In this study the different extracts tested showed less inhibition of acute inflammation than methanolic extract. The methanolic extract was further used in different doses and the anti-inflammatory efficacy was found to be dose dependent. The results obtained were significant with the control and the standard groups. In the arthritic model the methanolic extract showed decrease in the paw volume of arthritic animals and also in the arthritic symptoms. Again the results obtained were found to be significantly dose dependent. From the results obtained it can be concluded that this extract may serve as a source of drug against the rheumatoid arthritis.

## 1. Introduction 

Rheumatoid arthritis (RA) is an autoimmune disorder characterized by synovial inflammation and irreversible joint destruction and leads to significant disability [1]. It has affected about 1% of the population throughout the world with male and female ratio of 1 : 2.5 [2]. The etiology of this disease is still unknown [3]. The various proinflammatory molecules including reactive oxygen species, prostaglandins, leukotrines and cytokines released by macrophages are involved in the cause of this disorder [4, 5]. The potential target for the treatment of chronic inflammatory conditions can be the regulatory checks of these mediators secreted by immune cells and the inhibition of enzymes like COX and LOX for the metabolic modulation of arachidonic acid [6, 7]. RA being a systemic disease can affect the whole body and internal organs (with exceptions) such as the lungs, heart, and eyes [8, 9].

Although different categories like nonsteroidal anti-inflammatory drugs (NSAIDs), immunosuppressants, and steroidal anti-inflammatory drugs are in use, the limitation is their potential side effects. The development of new safe, potent, less toxic antiarthritic drug is the growing concern all over [10, 11]. The remedy for arthritis cannot be thought beyond nature. A number of herbs are there that synergistically work to reduce chronic joint inflammation, such as rheumatoid arthritis and osteoarthritis [12]. Plants are one of the economic sources of chemical intermediates needed for the production of a number of well-established and important drugs [13]. The ancient texts have mentioned about 500 plants in the treatment of arthritis, but so far very less number of plants have been scientifically evaluated [14]. The drugs currently in use have at least about 25 percent which are partially derived from plants [8].

A large number of medicinal plants have been tested and found to contain active principles with curative properties against arthritis [15]. The current study was carried out on* Gentiana kurroo* Royle belonging to the family Gentianaceae which is a critically endangered (CR) medicinal plant species, endemic to the north-western Himalayas. The drug (rootstock) of this plant is administered in fevers and urinary complaints and also used as a bitter tonic, antiperiodic, expectorant, antibilious, astringent, stomachic, antihelminthic, blood purifier, and carminative [16]. The methanolic root extract of this plant contains tannins, alkaloids, saponins, cardiac glycosides, terpenes, flavonoids, phenolics, and carbohydrates and has been found to have the analgesic activity [17]. The ethanolic extract of the flower tops of this plant contains alkaloids, flavonoids, glycosides, free phenols, and sterols/ terpenes and thus have been shown the anti-inflammatory activity [18].

The current study was carried out to observe the effect of this medicinal herb on the acute and chronic inflammation and hence to evaluate its antiarthritic potential.

## 2. Materials and Methods

### 2.1. Collection and Identification of Plant Material

The plant material for* Gentiana kurroo* Royle was procured from subalpine region in Dachigam, identified in the Centre of Plant Taxonomy (COPT), Department of Botany, University of Kashmir. The specimen was retained in the herbarium of COPT vide voucher number 1804-KASH.

### 2.2. Preparation of Extracts

The whole plant material was cleaned, cut into small pieces, and shade dried. The plant material was pulverized into coarse powder and extracted successively using petroleum ether, ethyl acetate, methanol, and water, respectively, by soxhlet extraction. The solvents were allowed to evaporate in a rotary evaporator at 40°–45°C and the extracts obtained were stored in a refrigerator at 4°C. The yields of the petroleum ether, ethyl acetate, methanol, and aqueous extracts were 5.6, 4.3, 5.3, and 4.2% (w/w), for* Gentiana kurroo*.

### 2.3. Animals

Male albino Wistar rats (*Rattus norvegicus*) (120–160 g ± 20 g) were used for anti-inflammatory study. The animals were housed under standard laboratory conditions and fed with standard pellet diet (Lipton India Ltd.) and water was given* ad libitum*. All experimental protocols and the number of animals used for the experimental work were duly approved by the Institutional Animals Ethics Committee (IAEC) of Indian Institute of Integrative Medicine (CSIR), Canal Road Jammu (CPCSEA registration number 67/ CPCSEA/99). None of the animals was sacrificed during the current study.

### 2.4. Carrageenin-Induced Paw Edema

Carrageenin-induced paw edema model [19] was utilized to assess the acute anti-inflammatory potential of the test samples. Animals were divided into different groups, each group with the same number of animals. One Group served as control, the other group was used as positive control, and rats in the rest of the groups were administered with plant extracts. All drugs were prepared in 1% tween 20 and given orally 45 min. before carrageenin injection. The dose volume selected was 1 mL/100 g, body weight of rat. Carrageenin was prepared in normal saline (1%) and 0.1 mL was injected into the subplantar region of left hind paw. The volume of both paws was measured with volume differential meter (520-R, IITC Life Science, USA) after 4 hrs with the volume of right paw taken as uninjected paw volume. Percent inhibition was calculated by taking mean of the difference of right and left paw edema, using the following formula:

(1) $$\begin{array}{c} \%\;\text{inhibition}=\frac{C-T}{C}\times 100, \end{array}$$

where *C* = mean edema in the control group and  *T* = mean edema in the treated group.

### 2.5. Adjuvant Induced Chronic Arthritis

The intradermal injection of dead mycobacteria (*M. tuberculosis*) suspended in liquid paraffin (Freund's adjuvant) in rats induced arthritis by producing chronic inflammation of the articulations in genetically predisposed animals [20]. A method described by Newbould [21] was used with some modifications. Male albino Wistar rats weighing 140–160 g were divided into seven groups. Group I served as control and was given normal saline with 1% tween 20. Groups II–VI were administered the plant extract/s in different concentrations (mg/kg, bw). Drugs were prepared in 1% tween 20. The dose volume given was 1 mL/100 g rat. Group VII served as positive control and was given diclofenac (20 mg/kg) as the standard drug for the treatment of inflammation. Dosing was started on day 1, 2 h before immunization of rats with heat killed* M. tuberculosis* (10 mg/2 mL) in complete Freund's adjuvant (CFA). The dose of immunization was 50 *µ*L in the subplantar region of right hind paw. Dosing was continued once a day for 14 days. The volume of both paws was measured with volume differential meter (520-R, IITC Life Science, USA) after 4, 7, 11, and 14 days with the volume of left paw taken as uninjected paw volume. Percent inhibition was calculated by taking mean of the difference of right and left paw edema, using the following formula:

(2) $$\begin{array}{c} \%\;\text{inhibition}=\frac{C-T}{C}\times 100, \end{array}$$

where *C* = mean edema in the control group and  *T* = mean edema in the treated group.

## 3. Results 

### 3.1. Acute Anti-Inflammatory Test

**Figure 1:**
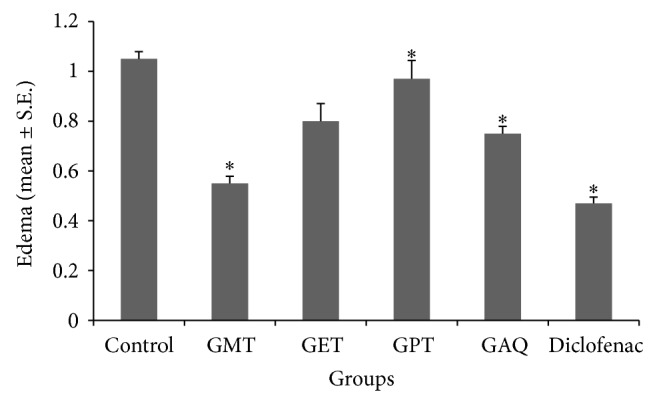
Graph showing effect on paw edema of Wistar ratsby different extracts of* Gentiana kurroo *Royle at a dose of 250 mg/kg (*n* = 4). ^*^ *P* < 0.05 (control versus treated groups); one-way ANOVA followed by Tukey's HSD test. GMT-methanolic extract; GET-ethyl acetate extract; GPT-petroleum ether extract; GAQ-aqueous extract.

Carrageenin-induced paw edema model was used to determine the acute inflammatory effect. Different plant extracts were screened for anti-inflammatory activity at a dose of 250 mg/kg bw. Methanolic fraction was found to have the maximum potential for suppressing the inflammatory response. The observed inhibitory effect in the paw edema was 47.62%. The results obtained in the methanolic fraction were found to be significantly (*P* < 0.05) related to control group (55.24%) (Figure 1).

Since methanolic fraction showed the maximum effect against inflammation, the same experiment was repeated with methanolic extract administered in different doses (50–750 mg/kg bw) orally to different groups. One group was kept as control and was given normal saline with 1% tween 20 in it. Diclofenac was taken as the standard drug and was administered to other groups at a dose of 20 mg/kg bw. Each group contained five animals. Animals taken for this study were male Wistar rats. The anti-inflammatory activity was seen by checking the decrease in paw edema after 4 h. The results showed increased dose-dependent activity of methanolic extract. At a dose of 750 mg/kg bw maximum activity was shown by methanolic extract which was even found to be higher than that of the standard drug. The inhibition in paw edema at this dose was 67.27% while the group which was given diclofenac showed 56.36% inhibition in paw edema (Table 1). The results were found to be statistically significant compared to control group but were found nonsignificant with standard group at *P* < 0.05.

### 3.2. Chronic Anti-Inflammatory Test

**Figure 2:**
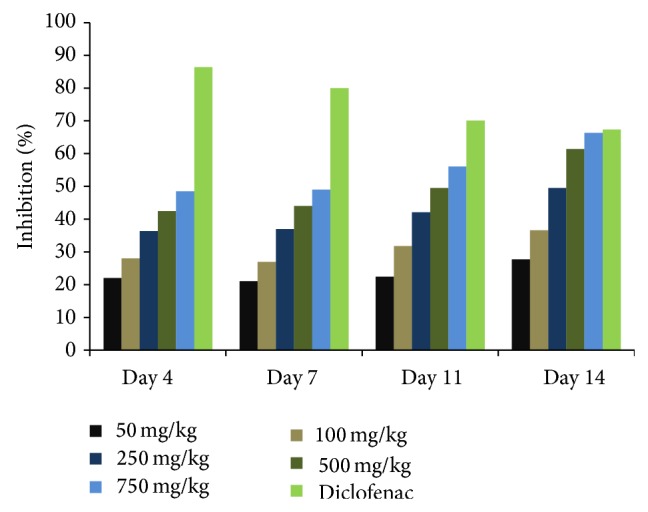
Dose and time dependent inhibition of paw edema in adjuvant arthritis with different concentrations of methanolic extract of* Gentiana kurroo*.

Experimental models have suggested that mycobacterial infections can trigger autoimmune arthritis, mainly through T-cell mediated responses. The methanolic extract was found to show activity against acute inflammation in the previous study. In order to see whether methanolic extract will have the same effect on chronic inflammation, adjuvant induced arthritis model was selected. For this study, male Wistar rats were taken in seven groups with each group having the same number of animals (*n* = 5). Arthritis was induced in rats by injecting dead mycobacteria in liquid paraffin (10 mg/2 mL; 50 *µ*L). Group I served as the arthritic control which was given normal saline with 1% tween 20. Groups II–VI were administered different doses of methanolic extract orally. Group VII was given diclofenac as the standard anti-inflammatory drug. Effect of the methanolic extract was observed by measuring the decrease in paw volume. Volume of both paws was measured after an interval of three days for fourteen days. The results showed a decrease in paw volume in animals administered with methanolic extract as compared to arthritic control. Paw volume taken was the average of difference between injected and uninjected paws. Uninjected paw showed edema formation after 11 days of mycobacteria immunization of animals. But the edema formation in treated groups was again found to be less as compared to arthritic control (Tables 2 and 3). Overall, the measurements showed dose dependent inhibition of edema in treated groups. As is obvious from Figure 2, the methanolic extract has dose as well as time dependent inhibitory effect on the edema formation. The results so obtained were found to be very significant as compared to arthritic control at *P* < 0.05.

## 4. Discussion 

In this study, the anti-inflammatory potential of different extracts obtained from* Gentiana kurroo* and their ability to reduce the arthritic symptoms have been investigated under* in vivo* conditions. Rat carrageenin paw edema was used as a model of acute inflammation and mycobacterium induced adjuvant arthritis as a model of chronic inflammation. The different extracts tested in acute inflammation showed anti-inflammatory effect with maximum potential shown by the methanolic extract. The methanolic extract was further analysed for the change in activity with the variation of dose. From the results obtained it was observed that increased methanolic doses showed increased anti-inflammatory activity. Most of the bioactive agents present in the medicinal herbs belong to secondary metabolites. These may be terpenoids or flavonoids in nature. As many monoterpenoids like camphene, borneol, and *β*-pinene are known to possess the anti-inflammatory property [22, 23]. Flavonoids like 6-methoxytricin have been seen to show the anti-inflammatory and analgesic activity [24]. The increased activity with increased doses may be because of the higher concentration of bioactive agent/s in this extract. The methanolic extract has shown more pronounced effect as compared to other extracts, which indicate the inhibition of chemical mediators of inflammation by the methanolic extract is more than the other extracts.

In adjuvant arthritis, the cellular immune response to mycobacterial antigens has been detected and is probably involved in the development of arthritis [25]. Identification of the 65 kDa protein as the main target of T-cell mediated responses [26, 27] and its cross-reactivity to purified cartilage proteoglycans [28] included in rheumatoid arthritis [29] has emphasized its importance in arthritis during mycobacterial infections. Since rheumatoid arthritis is an autoimmune disorder and involves the inflammation of joints, the adjuvant arthritis model also features the same symptoms. In our previous study the methanolic extract was found to show immunosuppressant activity [30]. So this arthritic model was used to explore the potential of methanolic extract. Now from the results observed it was found that the extract treated arthritic animals showed decreased inflammation of joints. The decrease in the paw volume was found to be consistent with the increase in dose. During the study it was also found that the development of arthritis was less in extract treated animals which was obvious from the inflammation seen in uninjected paw. In control group the uninjected paw was found to have high inflammation after 14 days, while in extract treated group and standard group the inflammation found was very much less. From these observations we find that the methanolic extract is showing the anti-inflammatory efficacy in both acute and chronic inflammation. This anti-inflammatory potential of methanolic extract may be because of the inhibition of proinflammatory cells [30]. This experimental work is just an attempt to establish* Gentiana kurroo *methanolicextract as an anti-inflammatory drug against rheumatoid arthritis. Further studies are carried for the possible mechanism and the identification of the bioactive compound from the extract.

## 5. Conclusion 

Results of the present study contribute towards the exploration of this herbal drug in the treatment of rheumatoid arthritis. However, no animal model completely depicts the pathophysiology and disease progression in this debilitating disease. Therefore, further investigational studies are required to elucidate the exact mechanism and the identification of the bioactive compound from this herbal extract. From the results obtained it can be concluded that this extract may serve as a source of drug against the rheumatoid arthritis.

## Figures and Tables

**Table 1:** Effect of methanolic extract of *Gentiana kurroo *on carrageenin-induced paw edema in Wistar rats (mean ± S.E) (*n* = 5).

S. number	Groups	Dose (mg/kg)	Initial paw vol. (mL)	Paw vol. after 4 h (mL)	Edema (4 h)	%age inhibition (4 h)
1	Control	NS	0.94 ± 0.024	2.04 ± 0.06	1.1 ± 0.055^a^	—
2	GI	50	0.96 ± 0.04	1.98 ± 0.1	1.02 ± 0.1^a^	7.27
3	GII	100	1.04 ± 0.068	1.94 ± 0.081	0.9 ± 0.083^a^	18.18
4	GIII	250	1.1 ± 0.045	1.68 ± 0.037	0.58^b^	47.27
5	GIV	500	1.02 ± 0.02	1.46 ± 0.068	0.44 ± 0.06^b^	60
6	GV	750	1.06 ± 0.051	1.42 ± 0.058	0.36 ± 0.081^b^	67.27
7	Diclofenac	20	1.04 ± 0.051	1.52 ± 0.037	0.48 ± 0.037^b^	56.36

Values along the same column with different superscripts are statistically significant to each other using Tukey's HSD test (*P* < 0.05).

**Table 2:** Effect of methanolic extract of *Gentiana kurroo* on mycobacterium induced adjuvant arthritis in 1st week.

Drug	Dose (mg/kg bw)	Body weight (g) 1st week	Edema (4 days)	Edema (7 days)
Control	NS	145.6 ± 1.29	2.64 ± 0.22	2 ± 0.13
GI	50	148 ± 3.16	2.06 ± 0.12	1.58 ± 0.21
GII	100	149.8 ± 3.14	1.9 ± 0.18^*^	1.46 ± 0.17
GIII	250	148.2 ± 3.07	1.68 ± 0.13^*^	1.26 ± 0.1^*^
GIV	500	150 ± 2.34	1.52 ± 0.17^*^	1.12 ± 0.11^*^
GV	750	148.2 ± 2.65	1.36 ± 0.15^*^	1.02 ± 0.04^*^
Diclofenac	20	151 ± 2.76	0.36 ± 0.09^*^	0.4 ± 0.06^*^

Values are represented as mean ± S.E. (*n* = 5). ^*^ *P* < 0.05 (control versus treated groups) using one-way ANOVA followed by Tukey's HSD test.

**Table 3:** Effect of methanolic extract of *Gentiana kurroo* on mycobacterium induced adjuvant arthritis in 2nd week.

Drug	Dose (mg/kg bw)	Body weight (g) 2nd week	Edema (11 days)	Edema (14 days)
Control	NS	156.2 ± 3.64	2.14 ± 0.09	2.02 ± 0.09
GI	50	156.4 ± 2.84	1.66 ± 0.17	1.46 ± 0.27
GII	100	158.2 ± 3.34	1.46 ± 0.28	1.28 ± 0.12
GIII	250	159.2 ± 3.38	1.24 ± 0.15^*^	1.02 ± 0.21^*^
GIV	500	158.6 ± 1.94	1.08 ± 0.16^*^	0.78 ± 0.28^*^
GV	750	160.2 ± 2.11	0.94 ± 0.07^*^	0.68 ± 0.09^*^
Diclofenac	20	159.4 ± 1.69	0.64 ± 0.06^*^	0.66 ± 0.15^*^

Values are represented as mean ± S.E. (*n* = 5). ^*^ *P* < 0.05 (control versus treated groups) using one-way ANOVA followed by Tukey's HSD test.
